# Intranasal sodium citrate in quantitative and qualitative olfactory dysfunction: results from a prospective, controlled trial of prolonged use in 60 patients

**DOI:** 10.1007/s00405-020-06567-7

**Published:** 2021-01-20

**Authors:** K. L. Whitcroft, N. Gunder, M. Cuevas, P. Andrews, S. Menzel, A. Haehner, T. Hummel

**Affiliations:** 1 Interdisciplinary Center for Smell & Taste, Department of Otorhinolaryngology, TU Dresden, Fetscherstrasse 74, 01307 Dresden, Germany; 2 Ear Institute, University College London, London, UK; 3 Centre for the Study of the Senses, Institute of Philosophy, School of Advanced Studies, University of London, London, UK

**Keywords:** Post-infectious olfactory dysfunction, Olfaction, Hyposmia, Functional anosmia, Treatment, Sodium citrate

## Abstract

**Objectives:**

We have previously shown that treatment with intranasal sodium citrate may be beneficial in post-infectious olfactory dysfunction. Sodium citrate reduces free intranasal calcium and is, therefore, thought to prevent calcium-mediated feedback inhibition at the level of the olfactory receptor. We aimed to determine whether treatment with a 2-week course of intranasal sodium citrate improves quantitative olfactory function in patients with post-infectious impairment. We also aimed to determine whether sodium citrate is beneficial in treating qualitative olfactory dysfunction.

**Methods:**

We performed a prospective, controlled study. Patients applied intranasal sodium citrate solution to the right nasal cavity for 2 weeks. The left nasal cavity was untreated and, therefore, acted as an internal control. Monorhinal olfactory function was assessed using the “Sniffin’ Sticks” composite ‘TDI’ score, before and after treatment. The presence of parosmia and phantosmia was also assessed.

**Results:**

Overall, there was a significant increase in TDI after treatment (using the best of right and left sides). Treatment with sodium citrate did not significantly improve quantitative olfactory function, compared to control. The proportion of patients reporting parosmia did not change significantly after treatment. However, there was a significant reduction in the proportion of patients reporting phantosmia, at the end of the study period.

**Conclusions:**

Treatment with intranasal sodium citrate for a period of 2 weeks does not appear to improve quantitative olfactory function in patients with post-infectious impairment, compared to control. It may, however, be beneficial in treating phantosmia, which should be further addressed in future work.

## Introduction

Olfactory dysfunction affects approximately 20% of the adult population [1, 2], and can cause significant impact on quality of life [3, 4]. At present, effective treatment options are limited, though previous work from our lab and others has demonstrated that use of intranasal calcium buffers may lead to short-term improvement in olfactory function [5, 6].

Free intranasal calcium is implicated in olfactory signalling through its role in the downstream signalling cascade of the olfactory receptor. Following activation by volatile chemicals, G-protein coupled olfactory receptors allow non-specific influx of cations into the olfactory receptor neuron through cyclic nucleotide gated cation (CNG) channels. In turn, sodium and calcium ion influx into the cell causes depolarisation and action potential generation [7]. However, rising intracellular calcium concentration also initiates downstream pathways involved in receptor feedback inhibition. These pathways involve reduction in cation flow through CNG channels, either via direct interaction with calcium–calmodulin [8–10], or by calcium-dependent phosphorylation of adenylyl cyclase [11–14]. Accordingly, it is speculated that reduced intranasal free calcium may in turn reduce feedback inhibition, resulting in amplified receptor response and improved olfactory function [15].

Sodium citrate is a calcium sequestrant. When applied intranasally, a small number of previous clinical studies have demonstrated improved quantitative olfactory function. Work from our lab showed that one-off application of sodium citrate improved short-term psychophysical test scores in patients with post-infective olfactory dysfunction (PIOD) [6, 16]. The aim of the present study was to determine whether prolonged use of intranasal sodium citrate improves quantitative and qualitative olfactory function in patients with PIOD. We therefore performed the following prospective controlled trial in 60 participants.

## Methods

### Study design

Prospective, controlled trial.

### Patient selection

Adult patients (> 18 years) with PIOD were recruited from the Smell and Taste Clinic at the Department for Otorhinolarnygology, TU Dresden. PIOD was defined as outlined in the Position Paper on Olfactory Dysfunction [17]. Patients with psychiatric, neurodegenerative or other conditions affecting olfaction were excluded.

### Olfactory assessment

Monorhinal quantitative olfactory testing was undertaken before and after the full course of treatment, using the “Sniffin’ Sticks” (SnSt) test battery (Burghart Messtechnik, Germany). This validated psychophysical tool allows for the assessment of odour threshold, discrimination and cued identification, with composite scores being used to diagnose quantitative olfactory dysfunction [18, 19]. For each of the individual subcomponents of the SnSt, as well as the composite score, higher values indicate better olfactory function. For detailed description of olfactory testing using the SnSt, please see ref [16]. Previous work has demonstrated that clinical improvement can be assumed where threshold score increases by ≥ 2.5, discrimination or identification scores increase by three points or composite TDI increases by ≥ 5.5 [20].

Qualitative olfactory dysfunction (presence of parosmia or phantosmia) was also assessed before and after the treatment period through use of structured patient history.

### Treatment regimen

All patients were treated with sodium citrate to the right nasal cavity (1 ml, 3.5 g/140 ml, pH 7.4, 298 mOsmol/L). This concentration was shown to be effective in our previous studies. The application was with a glass pipette (‘dropper’). Patients were instructed to apply the medication whilst lying in the ‘Kaiteki’ position, lying on the right side with neck turned laterally away from the bed by 20–30° and neck extended 20–40°. Following application, they were instructed to maintain this position for 1–2 min. Ten to fifteen drops were used in total. Patients continued treatment in this way two times per day for a period of 2 weeks. The contralateral nasal cavity was used as an internal control and, therefore, did not receive treatment. Our reason for using the contralateral nostril as control was threefold: 1—use of an ‘internal’ control in this way reduces systematic differences between treated and non-treated groups and thereby reduces the risk of such differences confounding our results; 2—use of an internal control in this way increased the efficiency of our study design; 3—we demonstrated temporary improvement in quantitative olfactory function following a single, monorhinal application of sodium citrate in our previous work; therefore, elected to keep this study design [21, 22].

Any potential side effects of the medication were recorded.

### Statistical analysis

Statistical analysis was performed using GraphPad Prism (vGraphPad Software, LaJolla). Parametric and non-parametric tests (Student’s *t *test/Wilcoxon signed rank test, Chi-squared test/Fisher’s exact test) were used as appropriate and statistical significance was assigned where *p* < 0.05. Results are given as mean ± standard deviation, unless stated otherwise.

## Results

### Patient demographics

In total, 60 participants with PIOD were recruited. Of these, 38 were female, 22 male and mean age was 61 years (range 25–85 years). The mean duration of olfactory dysfunction was 244 ± 238 days and mean time between testing was 23 ± 13 days. Mean composite TDI score across all patients before treatment was 19.09 ± 6.31 (using best of two monorhinal scores, assuming that the better nostril determines overall olfactory function [23, 24]). Again using the best of two monorhinal scores, 37 participants were hyposmic and 23 anosmic at baseline.

### Quantitative olfactory function

**Fig. 1:**
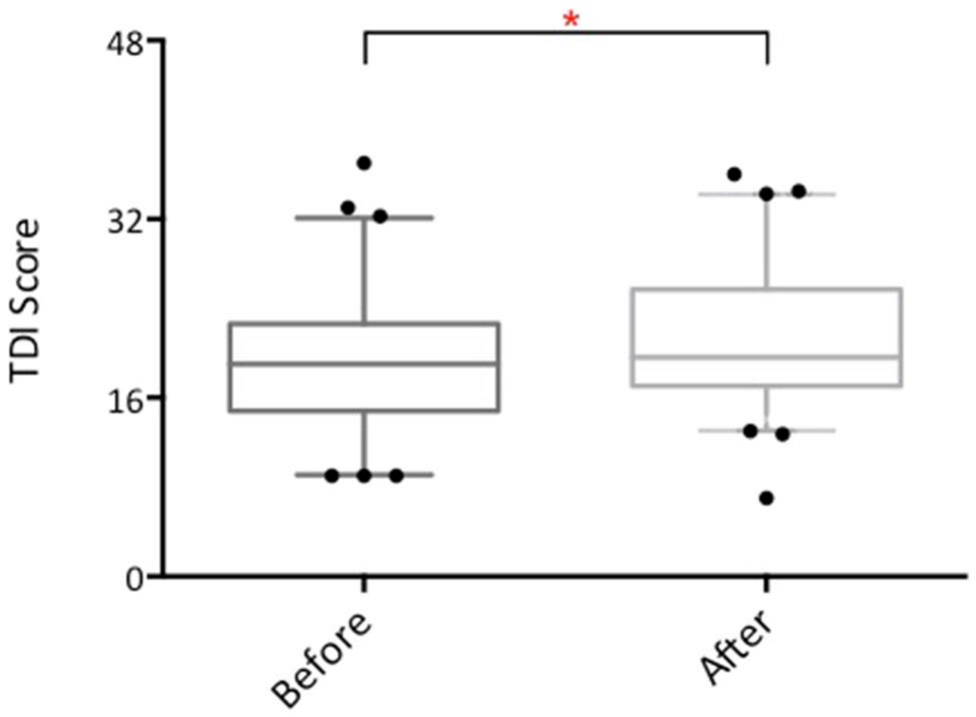
Box and whisker plot showing TDI scores before and after treatment with intranasal sodium citrate. *Error bars* 5–95% interval

Across all patients, there was a significant improvement in TDI score after treatment (comparing best pre- with best post-treatment TDI of right and left monorhinal scores; improvement of 2.08 ± 3.82 points, *p* < 0.0001, see Fig. 1). However, this improvement did not reach clinical significance (taken as ≥ 5.5 points).

**Fig. 2:**
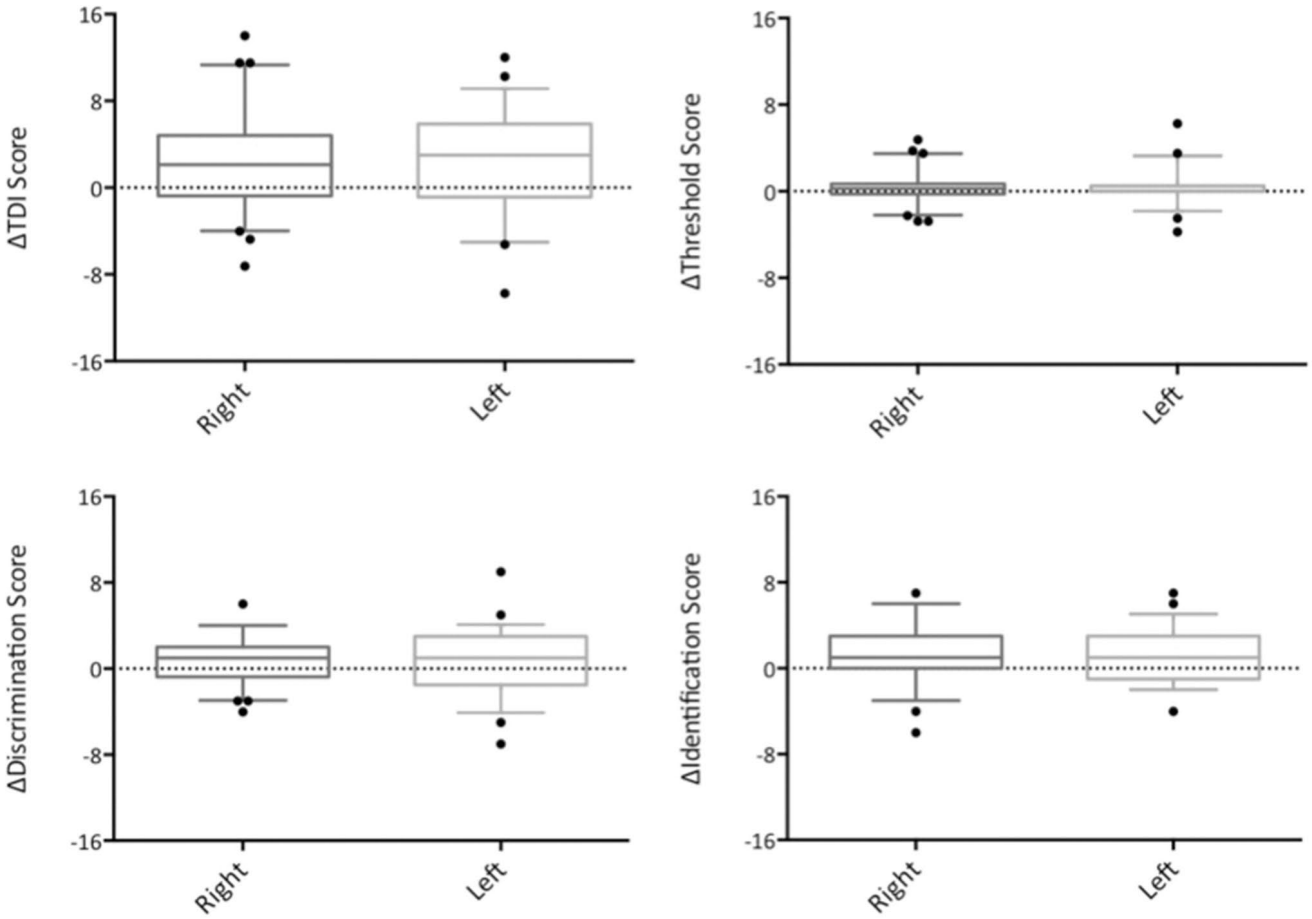
Box and whisker plots showing change in TDI score (top left), threshold score (top right), discrimination score (bottom left) and identification score (bottom right) before and after treatment with intranasal sodium citrate (right) or no treatment (left). *Error bars* 5–95% interval

To determine whether sodium citrate treatment led to significant improvement in quantitative olfactory function, we compared the change in olfactory test score [ΔTDI/T/D/I score = (TDI/T/D/I score after treatment)−(TDI/T/D/I score before treatment)] between right (treated) and left (untreated) sides. Accordingly, we found no significant difference between treated and untreated sides with respect to TDI, T, D and I (Fig. 2). This was true across all patients, as well as in subgroup analysis of hyposmic and anosmic patients.

**Fig. 3:**
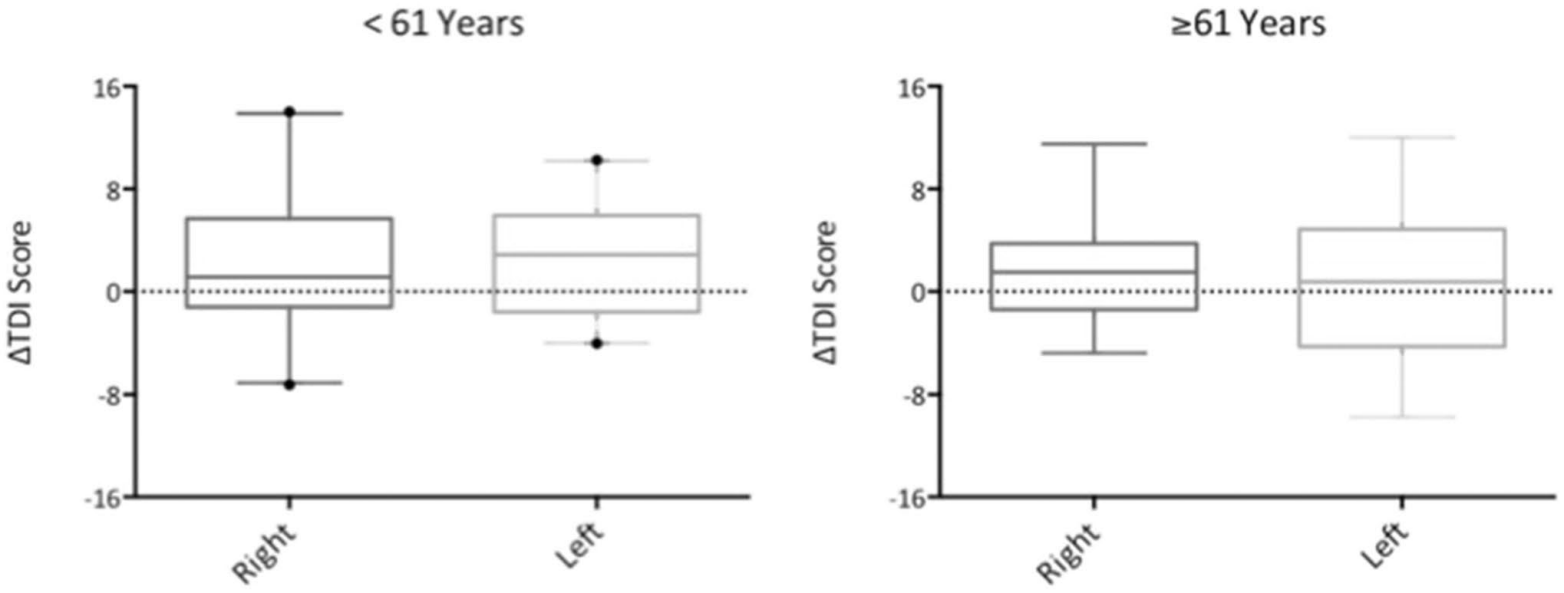
Box and whisker plots showing change in TDI score before and after treatment with intranasal sodium citrate (right) or no treatment (left), in patients under 61 years (left) or 61 years and over (right). *Error bars* 5–95% interval

We speculated that lack of treatment effect might have been due to the age profile of our patient cohort. To determine whether age related factors might have confounded potential treatment effect, we performed a further subgroup analysis according to age. We, therefore, divided our cohort according to age into two groups: patients below 61 years and patients aged 61 and older (61 being the median age in our cohort). There was a trend towards greater increase in TDI (using best of two monorhinal scores) after treatment in younger patients compared with older patients, however, this did not reach statistical significance (mean increase in TDI score 4.89 ± 11.75 in patients under 61 and 3.24 ± 10.75 in patients aged 61 and over, *p* > 0.05). When comparing change in monorhinal test scores, there was no significant effect of treatment in young or older cohorts. Additionally, there was no significant treatment effect when patients were divided into hyposmic and anosmic subgroups, in both the younger and the older cohorts (see Fig. 3).

### Qualitative olfactory function

At baseline, 23 patients reported parosmia and 17 phantosmia. Within the hyposmic subgroup, 18 reported parosmia and 12 reported phantosmia at baseline. After treatment, 12 reported parosmia, 1 phantosmia. Within the anosmic subgroup, 5 reported parosmia and 5 phantosmia at baseline. After treatment, 5 reported parosmia and 2 phantosmia. Within the under 61 age group (irrespective of quantitative function), 16 patients reported parosmia and 9 phantosmia at baseline. After treatment, 11 reported parosmia and 1 phantosmia. In patients 61 years or over (again, irrespective of quantitative function), 7 reported parosmia and 8 phantosmia at baseline. After treatment, 4 reported parosmia and 2 phantosmia.

**Fig. 4:**
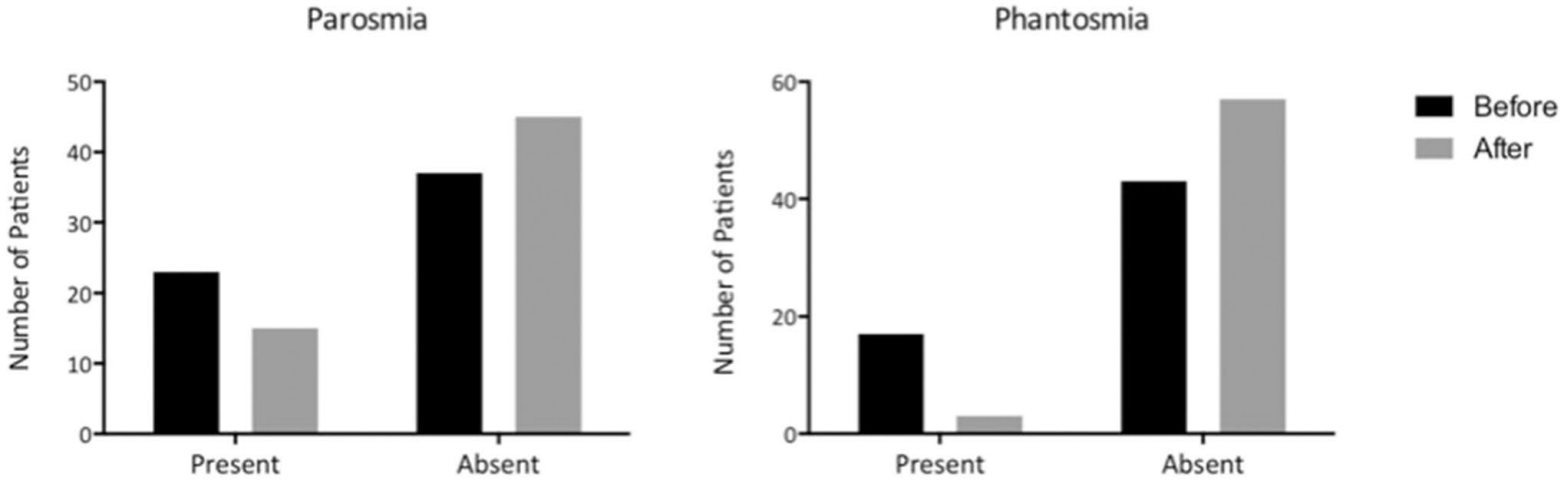
Bar charts showing number of patients experiencing parosmia or phantosmia before and after right-sided treatment with intranasal sodium citrate. The reduction in proportion of patients with phantosmia after treatment was statistically significant (*p* = 0.001)

There was a reduction in the overall proportion of patients reporting parosmia after treatment that did not reach statistical significance (23 patients before and 15 after treatment; *p* = 0.17). However, there was also a reduction in the proportion of patients reporting phantosmia after treatment, which did reach statistical significance (17 before and 3 after treatment, *p* = 0.001) (Fig. 4).

Subgroup analysis was also performed according to baseline quantitative olfactory function (hyposmia or anosmia) and age (< 61 or 61 +). Within the hyposmic subgroup, the reduction in proportion of patients reporting parosmia after treatment did not reach statistical significance (18 before and 10 after treatment, *p* = 0.055). However, the reduction in the proportion of hyposmic patients reporting phantosmia was statistically significant (12 before and 1 after, *p* = 0.00078). Within the anosmic subgroup, there was no reduction in proportion of patients with parosmia after treatment. Furthermore, the reduction in proportion of patients reporting phantosmia (5 before, 2 after) was not significant. Within the < 61 cohort, the reduction in proportion of patients reporting parosmia after treatment was not statistically significant (16 before and 11 after, *p* = 0.17). The reduction in number of patients reporting phantosmia, however, was statistically significant (9 before, 1 after, *p* = 0.013). Within the 61 + cohort, again the reduction in proportion of patients with parosmia was not statistically significant (7 before, 4 after treatment). The reduction in proportion of patients with phantosmia after treatment was statistically significant (8 before, 2 after, *p* = 0.039).

### Medication side effects

Medication side effects were as follows: sneezing (*n* = 12), nasal irritation (*n* = 11), pharyngeal irritation (*n* = 5), nasal obstruction (*n* = 1), increased nasal patency (*n* = 13). No side effects were sufficiently severe to necessitate discontinuation of therapy.

## Discussion

The results of this prospective controlled trial demonstrate that treatment with intranasal sodium citrate for a period of 2 weeks does not significantly improve quantitative olfactory function but does lead to a significant reduction in the proportion of patients reporting phantosmia.

### Quantitative olfactory function

Previous studies have demonstrated improvement in short-term quantitative olfactory function following one-off application of sodium citrate. In 2005, Panagiotopoulos and colleagues demonstrated statistically significant improvement in odour identification scores following one-off application of sodium citrate in their cohort of 31 patients with olfactory dysfunction of mixed cause [15]. However, this study design incorporated frequent testing with the 12-item screening version of the SS identification test—which was performed every 15 min after instillation of the medication. Due to this frequency of testing, it is possible that cue learning may have confounded the results obtained. In 2017, we performed a placebo-controlled trial in which sodium citrate was endoscopically applied to the right or left olfactory cleft, with physiological normal saline being applied as placebo control to the contralateral side. Fifty-seven patients with olfactory loss of mixed cause were recruited, and similar to Panagiotopoulos, we found a statistically significant improvement in odour threshold scores. However, this was limited to the subgroup of patients with PIOD (7 patients, mean improvement 2.29 ± 1.89, *p* = 0.02) [16]. Given the small participant number in this group, we performed a further prospective, placebo-controlled trial in which sodium citrate again was applied monorhinally, with physiological sodium chloride applied to the contralateral cavity, in 49 patients with PIOD [22]. Following one-off application with the ‘squirt device’, we found a statistically significant improvement in composite threshold + identification odour scores following treatment (mean improvement 0.87 ± 2.68 points, *p* = 0.04), but no significant improvement in individual threshold or identification scores alone. In both of these studies, treatment-related improvements in olfactory test scores reached statistical, but not clinical significance. For this reason, we speculated that prolonged use of intranasal sodium citrate might lead to clinically significant improvements in quantitative olfactory function.

In our current study, overall mean TDI score (taken as best of two monorhinal scores) improved (ΔTDI = 2.08 ± 3.82, *p* < 0.0001) following 2 weeks of daily use. Again, this improvement reached statistical, but not clinical significance. Furthermore, we did not find a significant improvement in monorhinal olfactory function following treatment, compared to control. This was true for change in TDI score, as well as change in the individual subcomponents (T/D/I), across all patients, as well as in hyposmic and anosmic subgroups, younger and older subgroups.

Several reasons for the lack of treatment effect on quantitative function seen in this study may be postulated. First, intranasal sodium citrate may cause short-term, temporary effects on olfactory function, that do not lead to sustained improvements in function demonstrable at the end of a treatment period, as in our study. This may explain the positive treatment effects seen in earlier studies, as compared with our present work. Second, whilst calcium is involved in downstream feedback inhibition of the olfactory receptor response, its role is not limited to inhibition, and is instead more neurobiologically complex. Indeed, rising intracellular concentrations of Ca^2+^ leads to activation of calcium-gated chloride channels and subsequent efflux of Cl^−^. This efflux modulates the excitatory response of the olfactory receptor, and potentiates cell depolarisation [25]. Furthermore, Ca^2+^ forms a significant proportion of the cation influx through CNG channels following olfactory receptor activation, and is therefore again involved in cell depolarisation [26]. It is possible that reducing intranasal free calcium over a prolonged period may result in equivalent results due to counterbalanced effects on depolarisation and inhibition.

### Qualitative olfactory function

Qualitative olfactory dysfunction, in the form of parosmia and phantosmia is relatively common in patients with PIOD [27]. Following monorhinal treatment with intranasal sodium citrate for 2 weeks, we demonstrated a significant reduction in the proportion of patients reporting phantosmia, but not parosmia.

Without treatment, idiopathic phantosmia is known to resolve slowly. In a retrospective study of 44 such patients, Landis and colleagues found that at 5 + years post diagnosis 38.8% had experienced complete resolution of their symptoms and 25% had experienced improvement in symptoms, whilst 38.7% reported no change and 4.5% had experienced deterioration [28]. Compared to these figures, we saw an 82.4% reduction in the number of patients with phantosmia after treatment with intranasal sodium citrate, over a period of just 2 weeks.

At present, treatment options for qualitative olfactory dysfunction are limited, and their mechanisms poorly understood. With regard to phantosmia, various modalities have been trialled, including haloperidol, sodium valproate, phenytoin, topiramate, verapamil, nortriptyline, gabapentin, topical cocaine, transcranial magnetic stimulation and surgical excision of the olfactory mucosa. A recent systematic review of the current literature concluded, however, that there was insufficient evidence or consensus to make recommendations on the management of this condition [29]. Of interest, the successful treatment cases described in this review generally involved longer time periods than the present study (from 18 months to 11 years).

In light of the significant reduction in reported phantosmia observed with sodium citrate therapy, we would speculate that this medication could be of use in treatment of such qualitative dysfunction. Previous work from Leopold and colleagues suggested that the pathogenesis of phantosmia may be related to peripheral dysfunction, at the level of the olfactory neuroepithelium [30]. This was based on histological analysis of neuroepithelium surgically excised from patients with phantosmia demonstrating changes such as reduced olfactory receptor neuron population; increased immature to mature neuron ratio; disordered axonal growth and presence of intraepithelial neuromas. It is possible that modification of receptor function through the application of sodium citrate may somehow mitigate the experience of phantosmia. However, it is unclear why the observed effect occurred following monorhinal application, and without significant effect on quantitative olfactory function. Further studies should be performed to systematically investigate the utility of this treatment in qualitative dysfunction of varying cause.

## Conclusions

During this prospective trial of intranasal sodium citrate therapy, we found no significant effect of treatment on quantitative olfactory function, compared to control. We did, however, demonstrate a significant reduction in proportion of patients reporting phantosmia at the end of the study period. Further research should investigative the effect of this treatment on qualitative olfactory dysfunction of varying cause.
